# Supplementary material for: Periodontal pathogens alter the synovial proteome. Periodontal pathogens do not exacerbate macroscopic arthritis but alter the synovial proteome in mice
Source: PLoS One. 2020 Dec 31;15(12):e0242868. doi: 10.1371/journal.pone.0242868 (PMC7774964; doi:10.1371/journal.pone.0242868)
Supplement: S1 File — (PDF) [file pone.0242868.s001.pdf]

S1 and S2 Fig. Periodontal pathogens alter the synovial proteome

Legend. The gut microbiomes exhibit robust stability. Neither induction of arthritis nor oral inoculation with *Pg* or *Aa* result in significant changes to alpha- and beta-diversity of the intestinal microbiome. These findings hold true for both experimental setups and are independent of whether periodontal disease or CIA are induced first. Stacked bars represent the proportion of bacterial families at given time points. The color code is presented below.

periodontal disease induced first

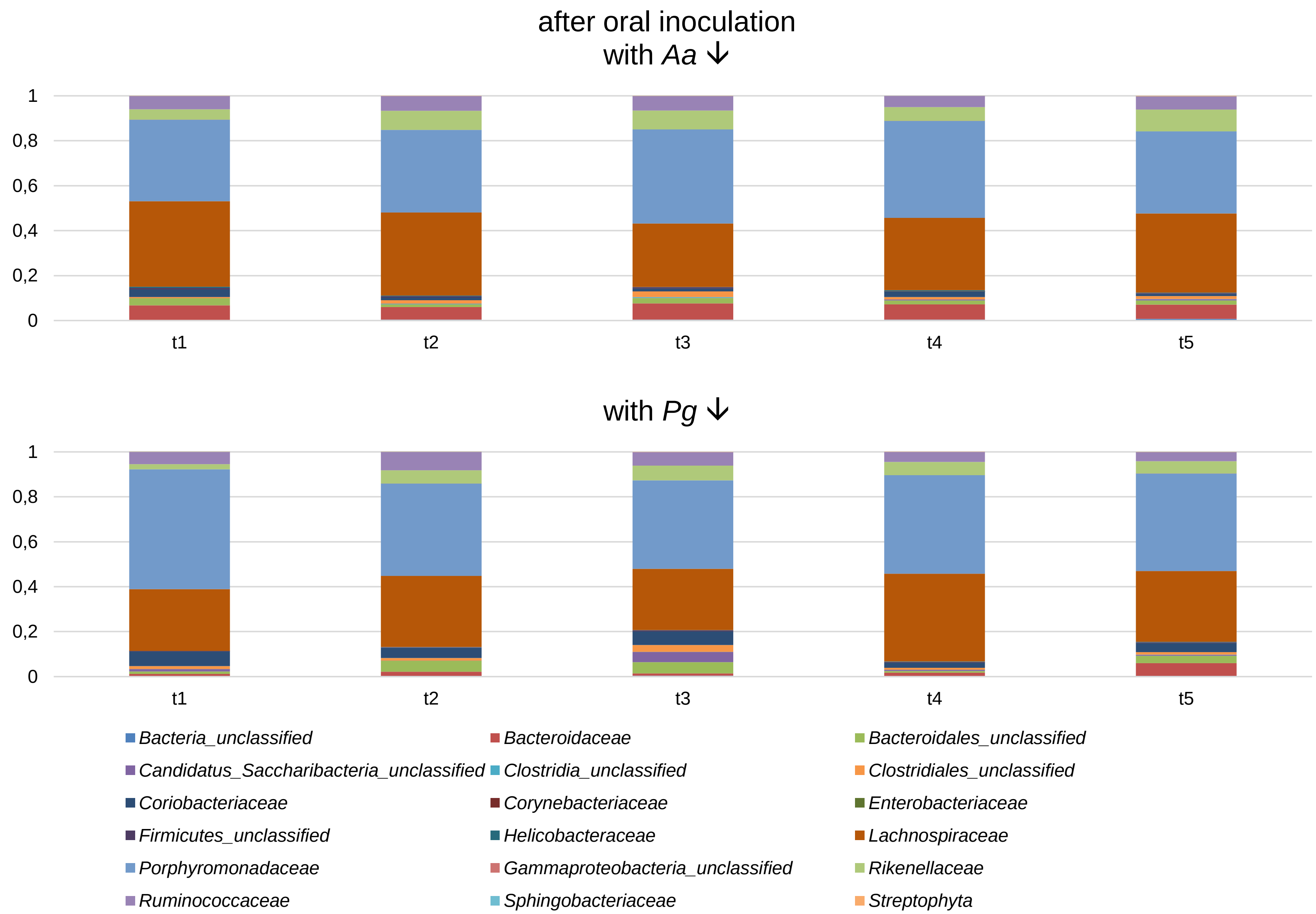

S1 Fig

CIA induced first

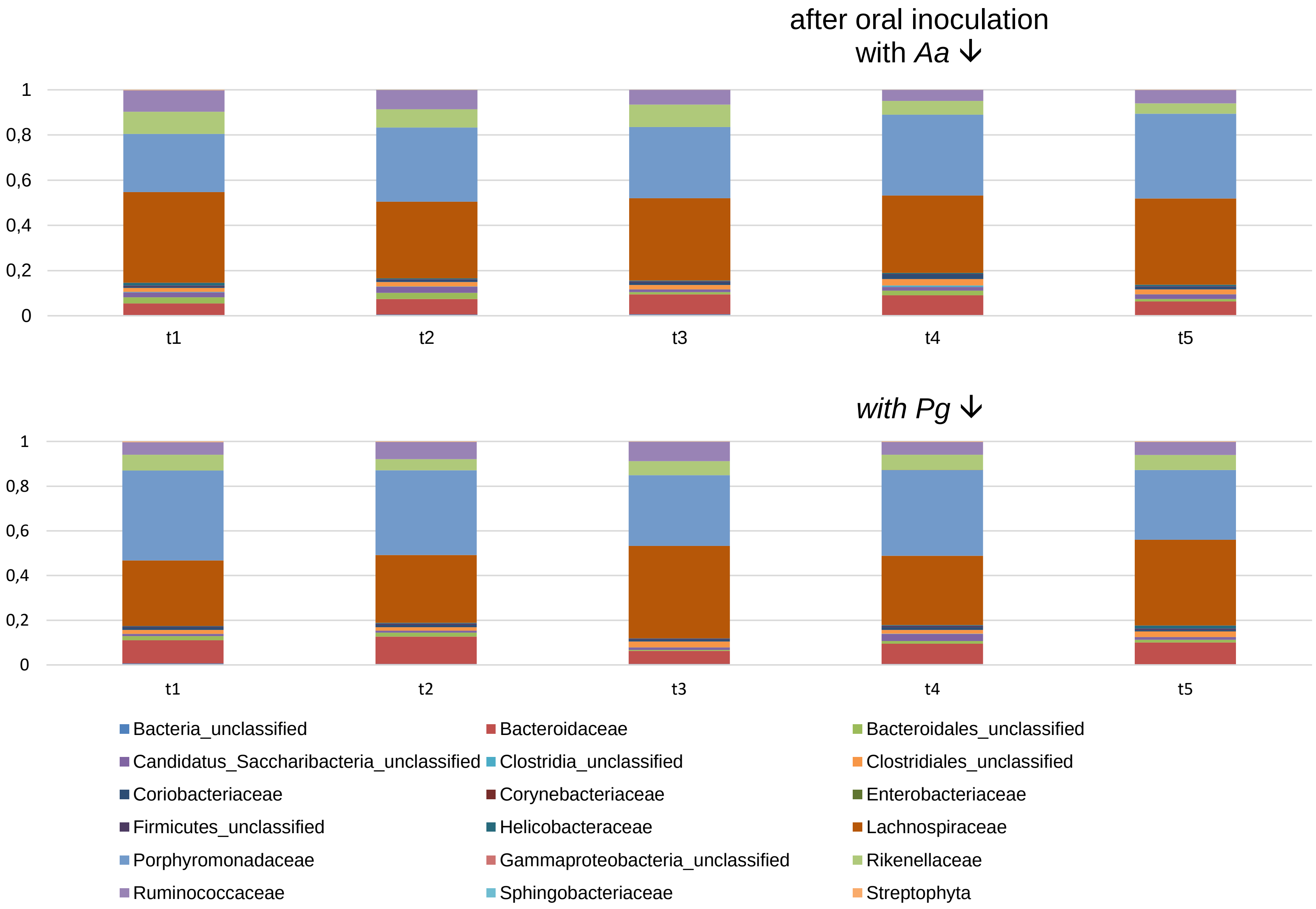

S2 Fig
